# Determination of mercury thermospecies in South African coals in the enhancement of mercury removal by pre-combustion technologies

**DOI:** 10.1038/s41598-020-76453-z

**Published:** 2020-11-06

**Authors:** Mpho Wendy Mathebula, Nikolai Panichev, Khakhathi Mandiwana

**Affiliations:** Department of Chemistry, Tshwane University of Technology, Arcadia, P.O. Box 56208, Pretoria, 0007 South Africa

**Keywords:** Environmental sciences, Chemistry

## Abstract

Samples of South African bituminous coals were analysed for total mercury (Hg) and Hg thermospecies concentrations using an RA-915 + Zeeman Mercury Analyser. Total mercury concentrations in samples of coals (n = 57) ranged between 10 ng g^−1^ and 493 ng g^−1^ with a mean value of 150 ± 53 ng g^−1^. Thermospecies of Hg were determined by monitoring Hg response as a function of sample temperature, increasing at 0.8 °C/s from ambient to 720 °C. This approach provides important information on thermal release of Hg species, as indicated by their appearance over specific temperature intervals. This permits identification of the presence of Hg thermospecies in coal and their quantification in each time (temperature) interval. It was found that 76% of tested bituminous coal samples release Hg species within low temperature intervals (20–180 °C and180–360 °C). The information generated in this study will aid in the selection of suitable coals for pre-combustion treatment that can lead to significant reduction of atmospheric Hg emission during coal combustion at power stations. This analytical approach can also be used for the creation of a system of coal classification based on the temperature of release of various Hg thermospecies.

## Introduction

The increasing concentration of mercury (Hg) in the environment, both from natural and anthropogenic sources, is a global problem that poses risk to the health of humans and wildlife^1–3^. Mercury is transported around the globe as gaseous elemental mercury (Hg^0^); therefore, its emission into the atmosphere poses hazardous consequences even in remote locations^4^. Exposure to mercury elicits a range of negative health effects^5^ and is recognized by the World Health Organization (WHO) as a harmful chemical due to its high toxicity, volatility and bioaccumulation^6^.

At present, the major sources of anthropogenic Hg emission are small scale gold production (SCGP) and coal combustion at electrical power stations and industrial boilers^7–9^. Mercury emissions from coal combustion take place mostly in the form of gaseous elemental mercury (Hg^0^), with small amounts of gaseous oxidized mercury (Hg^2+^), and particulate-bound mercury (Hg_p_). Methyl mercury is the most toxic organic mercury compound, commonly accumulating in fish and via the food chain can accumulate in mammals and humans to a very high toxic level. The most serious case of MeHg poisoning from contaminated fish took place in 1956 in Minamata, Japan^10^. The MeHg poisoning of humans, known as Minamata disease, united the world community in an effort to reduce global Hg pollution by creation of the United Nations Minamata Convention on mercury^11^. The role of the Minamata Convention is to protect human health and the environment from anthropogenic emissions that release mercury and mercury compounds to the atmosphere. Signatories of the Convention, which include major industrialized countries, such as China, USA, European Union (EU), Russia, India, Brazil and South Africa, have committed themselves to play a major role in this reduction program^12^.

Data on the inventory of global mercury emissions for 2015 show that approximately 5000 t of Hg originated from natural sources whereas 2220 t arose from anthropogenic sources. Artisanal and small-scale gold mining (ASGM) contributed 725 t (32.7%) and coal combustion, mostly from electricity generation, contributed 474 t (21%) of the annual anthropogenic discharge^13^. The highest amount of Hg entering the atmosphere from coal combustion (250 t) took place in China^14^.

Coal combustion during electricity generation is the main source of Hg emission to the South African atmosphere^15^. Exact data on the amount of Hg emitted was acquired only after several controversial publications. Thus, according to Pacyna et al*.*^16^, the amount of emitted Hg in South Africa in 2000 was 50 t. Recalculated data reported by Dabrowski et al*.*^17^ showed a much smaller number (9.8 t) but data reported by Masekoameng et al*.*^18^ was three-fold higher (29.47 t). Garnham and Langerman (2016)^19^ subsequently showed that the amount of Hg from coal combustion in South Africa could range between 16 and 20 t/annum. Such differences may arise simply from uncertainty in the results of analytical determinations of the Hg content in coals.

In order to reduce Hg emissions from coal combustion, it was recommended that only coal having lower Hg content should be used^20^. Reduction during coal combustion is possible by preliminary cleaning, either by washing or by thermal treatment prior to combustion^21–25^. Coal washing removes the Hg associated with incombustible mineral materials such as pyrite (FeS_2_) and thus reduces ash content to improve coal heating value. Some coals contain large amounts of Hg associated with their organic fraction and this can be a setback as it cannot be removed by coal washing. Therefore, controlled pyrolysis of coal provides a more promising alternative for mercury removal from all types of coals prior to combustion. However, the rate of mercury removal is affected by the treatment temperature, heating time, sweep gas flow and especially by the forms of mercury present. The efficiency of such thermal cleaning will be greatest in coal containing highly volatile Hg species. Mercury compounds that decompose at high temperature require much higher energy to release Hg species from coal. Such coal should be characterized in advance of its combustion to identify the most suitable pre-treatment temperature for removal of Hg^26,27^.

South Africa, as a signatory to the Minamata Convention is required (under Article 8), to reduce Hg emissions from coal-fired power plants during electricity generation^11,28^. The present study was initiated to develop analytical methods capable of identifying coals that contain Hg species having low volatilization temperatures such that they can undergo appropriate pre-combustion treatment. A new coal classification system could also be created based on the temperature of release of various Hg species. Such a classification system would be useful in identifying the effective pre-combustion and pre-gasification coal treatment method for reduction of Hg in coal prior to its combustion.

## Experimental

### Instrumentation

A RA-915 + Zeeman mercury analyzer (Lumex, St. Petersburg, Russia) was used for the quantification of various Hg species in coal samples. The working principle of the Zeeman mercury analyzer has been detailed by Sholupov et al. (2004)^29^ and is based on the thermal desorption of Hg from solid coal samples followed by the detection of Hg atoms by atomic absorption. Background absorption is eliminated by the use of a high frequency Zeeman correction system^30^. The concentration of Hg in the sample is determined from a calibration curve plotted as integrated analytical signal (arbitrary units) versus absolute mass of Hg (ng). Real-time measurement of Hg during its thermal release from samples is accomplished within 60–100 s at a resolution of one second (1 s).

### Samples

Samples of coals (n = 57) were collected from coal mines of Gauteng, Limpopo, Mpumalanga, Free State, KwaZulu-Natal and Eastern Cape Provinces of South Africa following a standard method for obtaining representative samples^31^. Coal production in South Africa is concentrated in the Highveld region of Mpumalanga Province where the Witbank, Highveld, and Ermelo coals are produced^32,33^. The mined coal is usually supplied to Eskom (Electricity Supply Commission) power stations for power generation and Sasol (abbreviated from Afrikaans Suid Afrikaanse Steenkool en Olie Maatskappy, literally translated as South African Coal and Oil Company in English) for use as feedback for the production of liquid fuel and chemicals and the surplus stock of coal is exported. The coal reserves in Witbank, Highveld and Waterberg (Limpopo Province) constitute approximately 70% of South Africa’s recoverable coal reserves^34^. The Witbank and Highveld coals are laterally contiguous as coal types as different characteristics coexist in the same coal beds.

### Analytical procedure for determination of total Hg

For the determination of total Hg, thermal decomposition of pulverized accurately weighted (to nearest mg) sub-samples was undertaken. The weighed sample was placed in a pre-cleaned quartz sampling boat and inserted into the furnace of the Hg analyser. The exact weight of the sample, ranging between 200 and 300 mg, was recorded using the RAPID software. Determination of total Hg was accomplished by heating samples until complete evaporation of Hg was ensured, usually within 60 to 100 s at 0.8 °C/s from ambient to 720 °C. Software permits temporal evolution of the analytical peak (Hg absorption) to be followed with the area under the peak, the maximum absorbance value and calculated concentration to be displayed. Each sample of coal was analysed in triplicate and results reported as a mean ± standard deviation of Hg concentration.

## Results

### Calibration of the mercury analyser

The mercury analyser was calibrated using certified reference materials (CRMs) containing Hg content in the range covered in the coal samples. Standard SARM 20 (MINTEK, South Africa, 250 ± 30 ng g^−1^) and PACS-2 (National Council of Canada, Canada, 3040 ± 99 ng g^–1^) were used for instrument calibration while CRM 7002 (Light sandy soil; Czech Republic, 90 ± 5 ng g^–1^) and CRM 024–050 (Loam soil 1, 710 ± 50 ng g^–1^) were used for method validation.. A calibration curve, plotted as integrated absorbance (peak area, arbitrary units) versus absolute mass of Hg (ng) defined by a typical regression equation such as $y = 350x - 71$ was obtained and used for the quantification of Hg. The calibration curve was linear (R^2^ = 0.998) up to 800 ng of Hg. This absolute value of 800 ng Hg indicates that the relative Hg concentration that can be determined in coal with acceptable precision and accuracy is 3200 ng g^−1^ for a sample mass of 250 mg. Therefore, this analytical approach based on thermal decomposition to determine the Hg content in coal is more favourable than chemical decomposition methods as it is more rapid and offers high efficiency.

**Figure 1:**
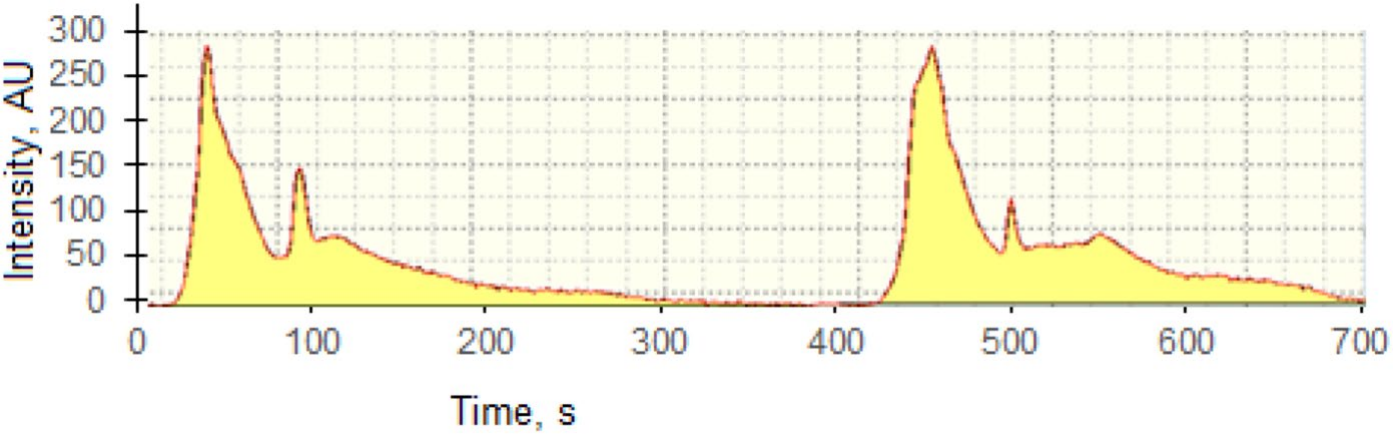
Reproducibility of Hg thermopeaks measurement.

In general, results for determination of Hg in the CRMs were in good agreement with certified concentration values at 95% level of confidence as the found values in SARM 20, PACS-2, CRM 7002 and CRM024-050 were 248 ± 7 ng g^–1^, 2990 ± 110 ng g^–1^, 94 ± 8 ng g^–1^ and 718 ± 14 ng g^−1^, respectively. To check the reproducibility of the analytical method, replicate samples of coal were analysed as shown in Fig. 1. The long-term results of Hg measurements (n = 33) were within the confidence intervals of certified values at the 95% confidence level.

### Limit of detection and limit of quantification

Due to a lack of “blank” coal samples on the market, the determination of the limits of detection (LOD) and quantification (LOQ) were calculated from the regression line of the calibration curve presented in a general form: $y = a + bx$. Numerical calculations were performed using the following formulas: $LOD = a + {{3S_{a} } \mathord{\left/ {\vphantom {{3S_{a} } b}} \right. \kern-\nulldelimiterspace} b}$ and $LOQ = a + {{10S_{a} } \mathord{\left/ {\vphantom {{10S_{a} } b}} \right. \kern-\nulldelimiterspace} b}$, where S_a_ is the standard deviation of the response y and b is the slope of calibration curve^35^. The LOD and LOQ were found to be 0.3 ng and 1.0 ng, respectively. These values indicate that for a coal sample of 250 mg the relative LOD is 1.20 ng g^−1^ and the LOQ is 4.0 ng g^−1^, illustrating that the methodology is capable of determining Hg content in coals of any commercial use.

### Influence of coal particles size on the results of Hg determination

The first factor to be evaluated during method development was the influence of particle size on the accuracy and precision of the results. This was achieved by analysing coal samples with particle sizes in the range 1–3 mm (1000–3000 µm) and pulverized coal with particle sizes of less than 50 µm. It was found that the results of the determination mercury in coals having a uniform distribution of Hg were not influenced by the particle size, as evident in Table 1.

**Table 1:** Results of Hg determination in coal of variable particle sizes, ng g^−1^.

Measurements number	Coal MH-3		Coal FS-5		Coal S2		Coal-G1	
	≤ 0.05 mm	1–3 mm	≤ 0.05 mm	1–3 mm	≤ 0.05 mm	1–3 mm	≤ 0.05 mm	1–3 mm
1	138	155	273	448	477	523	433	390
2	143	141	255	151	468	506	430	425
3	147	129	279	253	480	413	434	466
4	144	132	248	145	475	458	429	416
5	135	137	290	265	482	482	432	388
6	136	145	285	233	488	435	435	450
7	145	143	256	402	458	518	428	472
Mean ± SD	141 ± 5	140 ± 9	269 ± 16	271 ± 116	475 ± 10	476 ± 43	432 ± 3	430 ± 34
RSD (%)	3.5	6.4	5.9	42.8	2.1	9.0	1.0	7.9

This conclusion follows from a comparison of values of the relative standard deviation (RSD), which in the case of large particle size, RSD ranged between 6.4% and 42.8%, while for samples with small particle size, it was 1%-5.9%. Coal samples with inhomogeneous distribution of Hg can be analysed with good precision (RSD = 6%) only if particle size is first reduced. For samples having particle size 1000–3000 µm or higher, an RSD of as high as 42.8% could be reached. Results of this study suggest that coal samples should be ground to a particle size of 50 µm or lower to achieve higher accuracy and precision for coal with any kind of Hg distribution.

### Influence of sample mass on quantification of Hg

**Figure 2:**
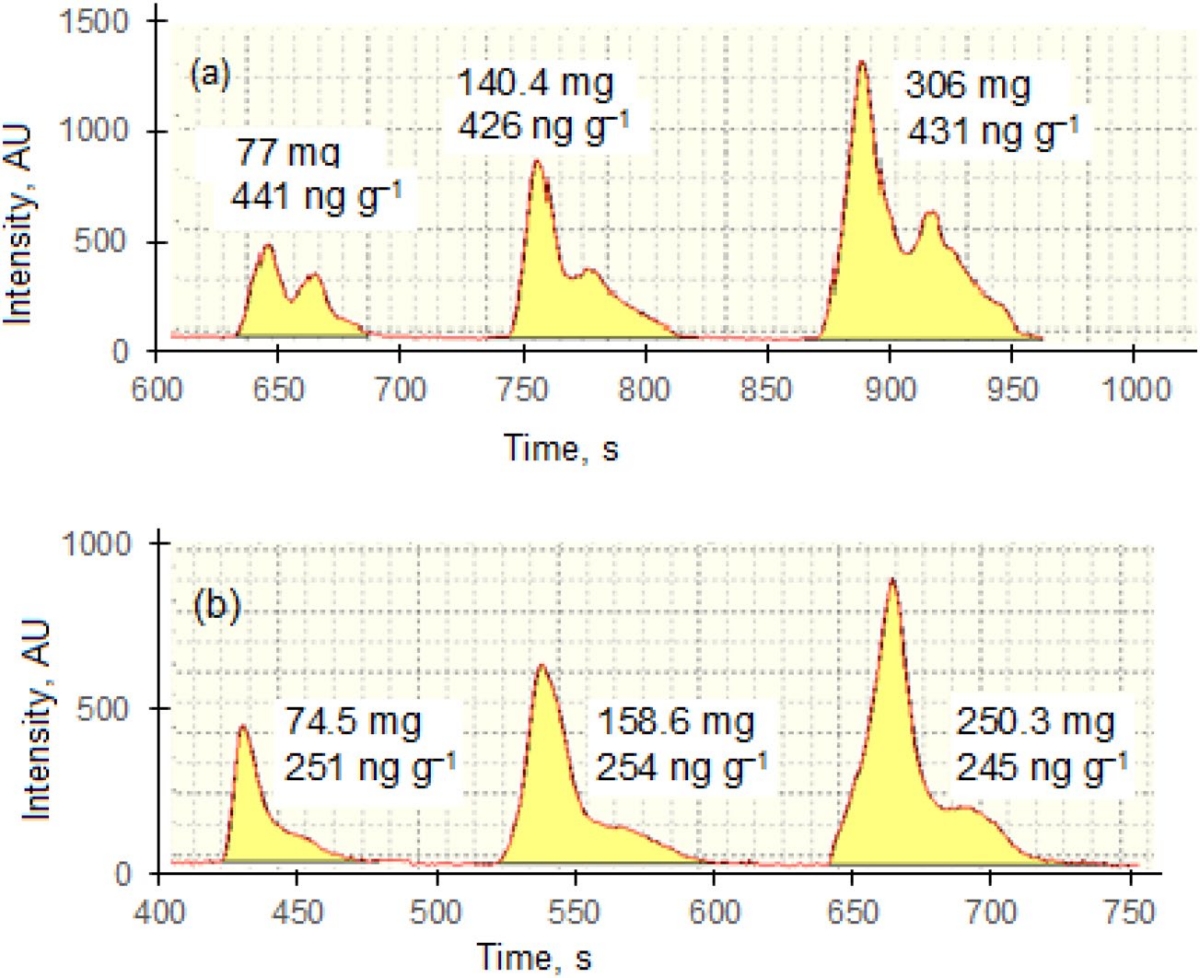
Results of subsample mass influence on Hg peak: **(a)** Coal G1; **(b)** SARM-20.

The second factor evaluated was the influence of sample mass on the results of determination of total Hg. For this purpose, coal standard SARM 20 and ordinary coal samples were used and the results are displayed in Fig. 2. For coal G1, masses of 77 mg, 140.4 mg and 305 mg yielded 441 ng g^−1^, 426 ng g^−1^ and 431 ng g^−1^, respectively, with mean concentration of 433 ± 8 ng g^−1^, irrespective of the sample mass taken for analysis. Similarly, for SARM 20, masses of 74.5 mg, 158.6 mg and 250.3 mg yielded 251 ng g^−1^, 254 ng g^−1^ and 245 ng g^−1^, respectively, with a mean Hg concentration of 250 ± 5 ng g^−1^ irrespective of rising sample masses. Similarly, quantitative results of Hg determination in four coal samples analysed in assessment of coal particle confirmed that sample mass of 50 mg to 300 mg has no influence on either the reproducibility of Hg concentration as shown by relative standard deviations that ranged between 1.0 and 4.2% (Table 2)**.** Therefore, it can be concluded that the results for determination of total Hg in coal are independent of sample mass taken for analysis.

**Table 2:** Results of Hg determination in coal samples of variable masses.

Mass of coal, mg	Concentration of Hg in coal, ng g^−1^			
	Coal MH-3	Coal FS-5	Coal S2	Coal-G1
50	143	280	510	436
100	139	264	480	428
150	148	270	485	433
200	140	277	471	430
250	145	272	491	439
300	138	269	455	426
Mean ± SD	142 ± 4	272 ± 6	480 ± 20	432 ± 5
RSD (%)	2.8	2.2	4.2	1.0

### Total Hg concentration in coals

Results for determination of total Hg in individual coals samples from South African Provinces, viz., Gauteng, Limpopo, Mpumalanga and KwaZulu-Natal and Free State, show high variability in concentration and range from 10 to 493 ng g^−1^ as shown in Table 3**.**

**Table 3:** Results of Hg determination in coals of South African Provinces.

Number	Province	Coal deposit	Number of samples	Concentration range, ng g^−1^	Mean ± SD, ng g^−1^
1	Limpopo	Waterberg	6	35–275	120 ± 95
2	Mpumalanga	Highveld	7	141–183	158 ± 22
3	Mpumalanga	Witbank	15	38–159	115 ± 60
4	Gauteng	Vereeniging	5	10–250	180 ± 150
5	Free State, Vaal	New Vaal	7	69–432	230 ± 298
6	KwaZulu-Natal	Vryheid	5	45–493	182 ± 42
7	Eastern Cape	Ecca	12	28–128	68 ± 33
Total			57	–	150 ± 53

The mean concentration varied from 68 to 230 ng g^−1^. Thus, the average Hg concentration in Mpumalanga’s coal fields of Highveld and Witbank were found to be 158 ± 22 ng g^−1^ and 115 ± 60 ng g^−1^, respectively. The mean Hg content in coal from Highveld deposit is in agreement with the average of 150 ± 50 ng g^−1^ reported by Wagner and Hlatshwayo^36^. The mean values of Hg content in coals Gauteng coal mines were found to be 180 ± 150 ng g^−1^ and compare well with the average of 200 ng g^−1^ found in Gauteng coals as reported by Bergh^37^. The lowest mean Hg content of 68 ng g^−1^ was determined in Eastern Cape coals and the highest mean Hg content of 230 ng g^−1^ was found in Free State Province. An abnormally high Hg concentration of 2514 ng g^−1^ was measured in one coal sample from Mpumalanga Province and is statistically different from all other results, but matches the result of 2430 ng g^−1^ measured in coal from Vryheid formation^38^. The mean value of Hg concentration in coal samples from all South African Provinces was found to be 150 ± 53 ng g^−1^.

### Total Hg in density fractionated coal samples

Density fractionated coal samples were analysed in order to identify the fraction of coal with reference to its specific gravity that has the highest Hg content. Two batches of selected coal samples from Witbank and Gauteng mines were used for study. Fractions of coals, separated by densities ranging between 1.40 g cm^−3^ and 2.00 g cm^−3^, were analysed for the Hg concentration and results summarized in Table 4.

**Table 4:** Total Hg determination in density fractionated coal samples.

Coal sample batch #	Type of Hg	Sample description	[Total Hg], ng g^−1^	Mean ± SD, ng g^−1^
Batch 1	Organic bound	WC-f1.40	817 ± 12	
		WC-f1.45	561 ± 20	583 ± 224
		WC-f1.50	371 ± 24	
	Clay bound	WC-f1.60	96 ± 1	50 ± 27
		WC-f1.70	71 ± 1	
		WC-f1.75	50 ± 2	
		WC-f1.80	49 ± 2	
		WC-f1.85	43 ± 3	
		WC-f1.90	23 ± 1	
		WC-f2.00	20 ± 1	
	Mineral bound	WC-Sink	79 ± 2	79 ± 2
Batch 2	Organic bound	GC-f1.4	44 ± 1	34 ± 11
		GC-f1.45	34 ± 1	
		GC-f1.5	23 ± 4	
	Clay bound	GC-f1.6	37 ± 1	26 ± 11
		GC-f1.7	26 ± 2	
		GC-f1.9	15 ± 1	
	Mineral bound	GC-Sink	1340 ± 35	1340 ± 35

*WC* Witbank coal, *GC* Gauteng coal.

For Witbank coals, the batch consisted of coal float samples, viz., WC-f1.40 to WC-fl2.0 and WC-Sink. It was found that low density float fractions contain more Hg (371 ± 24 ng g^−1^, 561 ± 20 ng g^−1^ and 817 ± 12 ng g^−1^) with total average of 583 ± 224 ng g^−1^ bound to organic fractions. Coal sink sample, WC-Sink, from this batch was found to contain only 79 ng g^−1^. For Gauteng coals, an average Hg content in coal float samples of low densities bound to organic compounds were found to be 34 ng g^−1^ while the mineral fraction of sink sample presented 1340 ng g^−1^. Such material was dominated by kaolinite minerals. These results show that the contribution of Hg bound to the organic fraction is significant compared to the total Hg content^39^.

### Results for the determination of thermospecies of Hg in coal.

**Figure 3:**
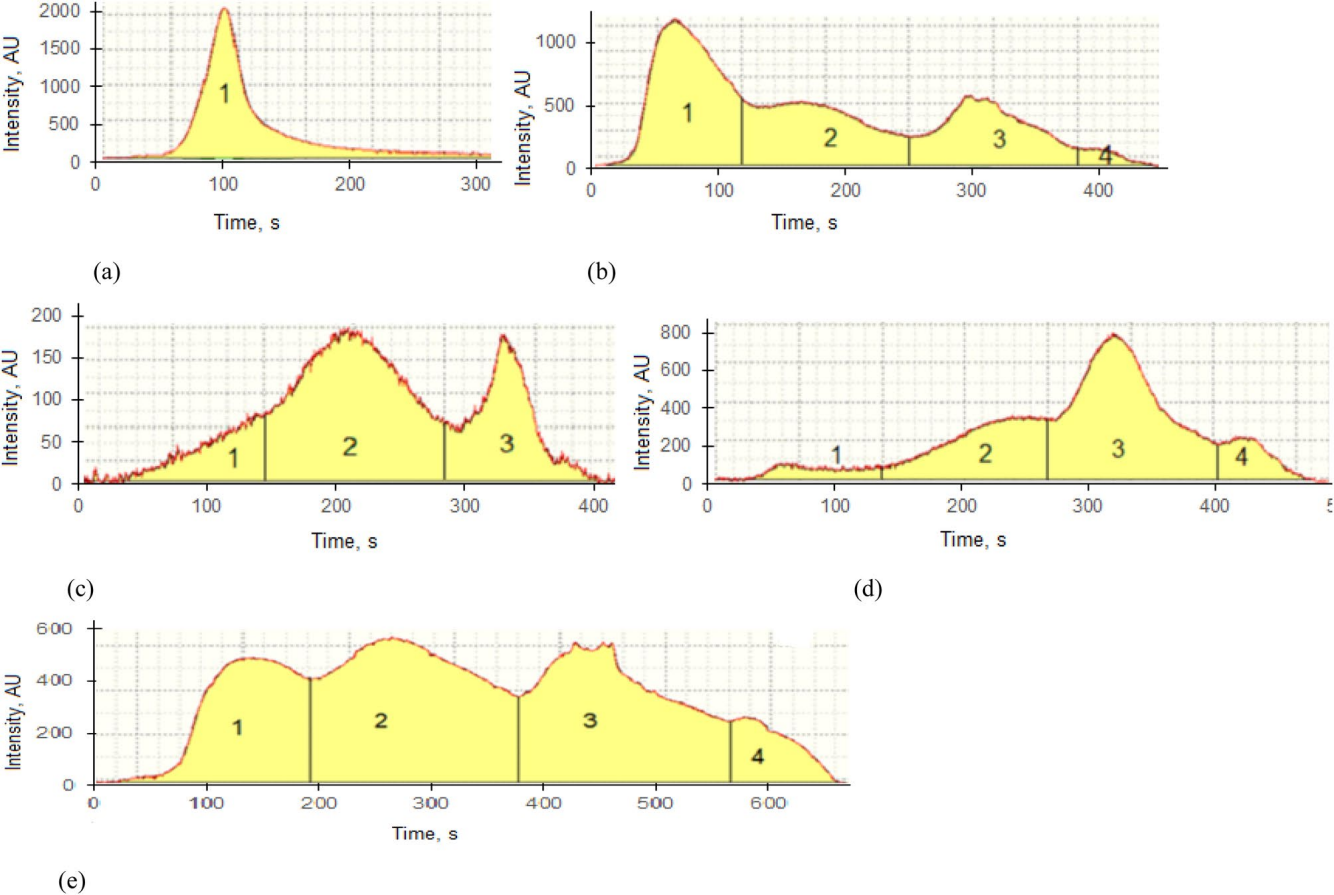
Examples of Hg thermospecies in South African coals: **(a)** coal with one low temperature thermo peak; **(b)** coal with multiple thermos species dominated with low temperature peak; **(c)** coal with three thermos peaks dominated with middle temperature peak; **(d)** coal with four thermospecies dominated with high temperature volatization; **(e)** coal with thermos species which volatile at all temperatures.

The procedure for determination of thermospecies of Hg developed by Mashyanov et al*.*, 2007^40^ demonstrated good reproducibility of results at over selected temperature intervals, as noted in Fig. 3. After analysis of all samples for the presence of Hg peaks, it was established that some coal samples may contain only one Hg species, as indicated by the presence of only a single peak over the full temperature range investigated, whereas other samples may contain two to four peaks that are associated with different chemical forms of Hg in the coal specimen (cf. Fig. 3). The total concentration (ng g^−1^) and percentage (%) of various thermospecies of Hg in selected samples from each of the South African provinces are shown in Table 5. These include coal samples with the smallest, close to the mean, and highest concentrations of Hg. It was found that out of twenty one (21) analysed bituminous coal samples (excluding pyrite coals and SARM 20), sixteen (16) coal samples or 76% coal samples contained thermospecies of Hg that are released in a low temperature interval of 180º to 360 ºC, suggesting that this is elemental Hg^0^ and/or Hg associated with organic compounds^41^.

**Table 5:** Concentration (ng g^−1^) and percentage (%) of Hg thermospecies in coals of South African Provinces.

Province and coalfield	Sample identity number	Total Hg, ng g^−1^	Hg thermospecies evaporated at specific temperature intervals, %			
			20 − 180 ºC	180 − 360 ºC	360 − 540 ºC	540 − 720 ºC
Limpopo, Waterberg	L1	38	100	–	–	–
	L2	106	8	75	15	–
	L3	148	26	72	2	–
Mpumalanga, Witbank	M2	15	100	–	–	–
	M6	150	34	66	–	–
	M14	467	23	43	23	11
Mpumalanga, Highveld	MH3	142	30	49	22	–
	MH5	165	30	52	20	2
	MH7	183	66	20	19	–
Gauteng	G2	35	100	–	–	–
	G4	174	88	12	–	–
	G5	210	25	65	10	–
Free State, New Vaal	FS1	66	94	6		–
	FS5	270	87	10	1	–
	FS7	448	75	13	11	5
KwaZulu-Natal	KZ2	32	100	–	–	–
	KZ3	100	78	20	2	–
	KZ5	114	85	6	14	–
Eastern Cape, Ecca	EC5	37	100	–	–	–
	EC8	74	55	45	–	–
	EC12	100	50	50	–	–
Selected samples	SARM 20	250	68	31	–	–
	Pyrite	542	41	24	31	3
	Pyrite sink	1366	47	28	18	7

## Discussion

Results of this investigation indicate that correct measurement of total Hg concentration in coal samples after thermal evaporation is constrained by achieving a number of conditions. One of them is the nature of the distribution of Hg in the coal which influence the reproducibility of determination of total Hg. In the case of a homogeneous distribution, the particle size is not an important factor, but if this is not satisfied and Hg is present in the form of minerals such as cinnabar (HgS) or other compounds high in Hg concentration, the RSD of results can be very high, leading to poor precision. Thus, analysis of coal samples having an uneven distribution of Hg present in particle sizes ranging from 1000 to 3000 µm could result in RSDs as high as 40%, whereas when using pulverized coal having particle sizes below 50 µm yields only 2% (cf Table 1). The extremely uneven distribution of Hg in samples from Vaal coal mine (northern Free State) could be connected with the geologic structure of the seam, wherein a combination of chemical and physical weathering has resulted in a highly undulating floor^33^. To achieve higher accuracy and precision, it is recommended that all coal samples be ground before analysis.

Another possible factor affecting reproducibility and accuracy of results during Hg determination in coal could be associated with sample mass taken for analysis. This study revealed that the Hg concentration in coal standard SARM 20 and coal G-1 derived from sample masses ranging from 50 to 300 mg were identical at the 95% level of confidence (Table 2). The mean total Hg in SARM 20 was determined to be 250 ± 5 ng g^−1^, in agreement with the certified value of 250 ± 10 ng g^−1^. The total concentration of Hg in coal G-1 was 432 ± 5 ng g^−1^, independent of sample mass in the range 50–300 mg, yielding an RSD of 1.0%. These data show that results for determination of Hg in coals are not influenced by mass of sample taken for analysis.

The last factor which can influence the results is the stability of Hg as regards evaporation from the samples. Stability of thermos peaks of Hg could be identified by the shape of resultant absorbance signals recorded during analysis (cf Fig. 1). Various shapes were obtained are shown in Fig. 3. They reflect the presence of different modes of occurrence of Hg in coals. Detailed studies of Hg thermo peaks indicate that coal contains various forms of Hg that are released over specific temperature ranges, viz., 20–180 ºC, 180–360 ºC, 360–540 ºC, 540–700 ºC, generating very stable and reproducible results, as summarized in Table 5.

The mean Hg concentration in coals of South African Provinces was found to be 150 ± 53 ng g^−1^ while the global average Hg content of coal, expressed on a whole-coal basis (the Clarke value for Hg in coal) is 100 ± 10 ng g^−1^ and is the same for bituminous, subbituminous, and lignite rank coals. This result confirms previous findings that concluded that the mean value of Hg in South African coals is higher than the global average^42^.

Analysis of density fractionated coal samples showed that such samples can be used to identify the coal fraction that concentrates the Hg (cf Table 4). In general, it was found that in some coal samples, Hg accumulates in organic compounds in low density coal fractions whereas in other samples of coal Hg is concentrated in the mineral sink and the highest content found was 1340 ± 35 ng g^−1^ in contrast to mineral bound coal fractionated sink sample of batch 1 (79 ± 2 ng g^−1^). The results of pyrolysis of the density-fractionated samples lead to the conclusion that the method of thermal speciation is more efficient for the selection of coal for Hg removal by mild pyrolysis before coal combustion. Thermospeciation of Hg in coal prevents direct application of pyrolysis technology to raw unsuitable coal with a high content of pyrite-bound Hg because the high pyrolysis temperature demands high energy consumption, thereby degrading overall coal heating efficiency.

In spite of the fact that most of South African coals are bituminous grade, 2% being anthracite grade and 1.6% being coal of metallurgical quality, several thermospecies of Hg were detected in most coal samples due to their different binding energy to coal matrix.

Coal may contain one or several Hg species that are released at variable temperatures (cf Table 5). The majority of such species were released in the range 20–180 ºC and can be attributed to elemental Hg as it is known to be vaporized in this range of temperature. The second dominant thermospecies, released between 180ºC and 360ºC, could be connected to the presence of organobound-Hg compounds, whereas the least amount of Hg is released between 360 and 540 ºC and may be connected with a pyrite bound Hg fraction^46^. Identification of the volatility temperature range of Hg in coal in advance of use is a necessary step required to select an appropriate method for removal of Hg during coal combustion for electricity generation.

## Conclusions

This study shows that the accuracy and precision of determination of total Hg in coals having an inhomogeneous distribution of Hg is dependent on the particle size of the samples taken for analysis. Particle sizes below 50 µm are recommended for analysis to ensure accurate and precise results. It was also found that the results of analysis are independent of the mass of the samples in the range of 50–300 mg. Results for determination of total Hg show that the mean value of Hg concentration in coal samples from all South African Provinces is on the level of 150 ± 53 ng g^−1^.

The presence of Hg species of various thermal stability in coal was demonstrated through detection of multiple desorption peaks, dominated by low temperature evaporation of elemental Hg released at 20–180 °C. The specific temperature range of Hg peaks creates a basis for selection of coals for preliminary cleaning prior combustion at electrical power stations according to the thermal stability of the Hg species. Identification of thermospecies is an analytical problem that requires further studies.

Analysis of density fractionated coal samples showed that Hg can be concentrated in both organic components of coals, as well as in mineral fractions. This study presents new insights into our knowledge of the forms of Hg present in South African coals.
